# Patient centered HCC surveillance - complementary roles of ultrasound and CT/MRI

**DOI:** 10.1007/s00261-024-04678-x

**Published:** 2024-11-11

**Authors:** Jason Heald, David T. Fetzer, Shuchi Rodgers, Vaibhav Jain, Alice Fung, Xiaoyang Liu, Stephanie Wilson, Aya Kamaya, Robert M. Marks

**Affiliations:** 1 Loma Linda University Medical Center, Loma Linda, USA; 2 The University of Texas Southwestern Medical Center, Dallas, USA; 3 Thomas Jefferson University Hospital, Philadelphia, USA; 4 University of Massachusetts Chan Medical School, Worcester, USA; 5 Oregon Health & Science University, Portland, USA; 6 University Medical Imaging Toronto, University Health Network, University of Toronto, Toronto, Canada; 7 University of Calgary, Calgary, Canada; 8 Stanford University, Stanford, USA; 9 University of California, San Diego, San Diego, USA

**Keywords:** Hepatocellular carcinoma, Surveillance, Ultrasound, Abbreviated MRI, LI-RADS

## Abstract

Hepatocellular carcinoma (HCC) is a leading cause of cancer-related mortality worldwide and is the fastest growing cause of cancer death in the United States (U.S.) In the U.S., current national clinical practice guidelines from the 2023 American Association for the Study of Liver Diseases (AASLD) Practice Guidance and the recently updated Liver Imaging Reporting & Data Systems (LI-RADS) Ultrasound (US) Surveillance v2024 core recommend semi-annual serum α-fetoprotein and US screening of patients deemed to be high risk for developing HCC. In this article, we will explore the transition to a patient-centered approach to HCC surveillance, including the role of the new LI-RADS US Surveillance v2024 core and the use of visualization score for determining ultrasound quality, the known risk factors for poor US image quality, and the potential options for alternative surveillance strategies when US may not be a viable option for certain patients, including multiphasic computed tomography (CT), magnetic resonance imaging (MRI), and several abbreviated MRI protocols.

## Background

Hepatocellular carcinoma (HCC) is a leading cause of cancer-related mortality worldwide and is the fastest growing cause of cancer death in the United States (U.S.), estimated to be responsible for approximately 30,000 deaths annually [1, 2]. HCC is the most common primary malignant liver tumor and is almost always found in patients with underlying chronic liver disease. Well-accepted high-risk factors include cirrhosis from any cause and certain populations with chronic hepatitis B virus (cHBV) infection. Other risk factors may include hepatitis C virus (HCV) infection, heavy alcohol consumption, metabolic-dysfunction associated fatty liver disease or steatohepatitis (MAFLD or MASH), obesity, diabetes, and male gender [3, 4]. Given the rising incidence of disease and high prevalence of the above risk factors, surveillance regimens including both biochemical testing and imaging surveillance play a key role in the management of patients with chronic liver disease.

In the U.S., current national clinical practice guidelines from the 2023 American Association for the Study of Liver Diseases (AASLD) Practice Guidance and the recently updated Liver Imaging Reporting & Data Systems (LI-RADS) Ultrasound (US) Surveillance v2024 core recommend semi-annual serum α-fetoprotein and US surveillance of patients deemed to be high risk for developing HCC [5, 6]. These guidelines define “high-risk” patients as those adults with cirrhosis of any etiology, and subsets of adult patients with cHBV regardless of the presence or absence of liver cirrhosis–These subsets include males over the age of 40 or females over the age of 50 from endemic countries, anyone from Africa over the age of 20, those with a family history of HCC, and those with a PAGE-B score greater than or equal to 10.

In this article, we will explore the transition to a patient-centered approach to HCC surveillance, including the role of the new LI-RADS US Surveillance v2024 core and the use of visualization score for determining ultrasound quality, the known risk factors for poor US image quality, and the potential options for alternative surveillance strategies when US may not be a viable option for certain patients.

## Diagnostic performance of US

The recommendations for the use of US for semi-annual surveillance of HCC are supported by data from a large prospective randomized controlled clinical trial, performed in China, that demonstrated a reduction in HCC mortality by up to 37% in screened high-risk individuals [7]. Other smaller studies have supported these findings [8]. Given the reduction in mortality, its high availability, lower cost, and excellent patient tolerability, US is positioned to serve as an ideal first-line option for imaging surveillance.

Ultrasound does, however, have some limitations, particularly for certain subsets of patients with significant hepatic steatosis or a markedly heterogeneous liver, and those with a large body habitus that limits sufficient acoustic penetration. This becomes relevant when considering the population in the United States which may not be represented by the study population of the aforementioned prospective Chinese study. Basing U.S. guidelines on results of the study from China is problematic because, unlike the current populations in America and other western nations, patients in the study had a low prevalence of underlying obesity and cirrhosis [7]. Recent studies show that alcohol and MASH-related cirrhosis, Child-Pugh Class C cirrhosis, and obesity are independent risk factors associated with worse sonographic visualization of the liver [9–11]. Additionally, two recent meta-analyses of 26 studies found that the pooled per-patient HCC sensitivity of US surveillance in patients with cirrhosis or chronic liver disease was 60–63% [12, 13]. However, it is important to note that despite one of the studies concluding that AFP provided no additional benefit to US in HCC surveillance [12], current data show that with the addition of semiannual serum AFP, as recommended in current surveillance guidelines, semi-annual US surveillance has similar performance to annual CT for the detection of early HCC in a surveillance population [14]. Additionally, the previously published performance data did not take visualization score into account, as now recommended in the updated LI-RADS US Surveillance v2024 core and AASLD Practice Guidance, and therefore likely underestimates the performance of US in many patients.

## Visualization scores - LI-RADS US Surveillance v2024 core

The LI-RADS US Surveillance v2024 core update aims to facilitate a patient-centered surveillance strategy by identifying those patients for whom US may not be the optimal surveillance imaging method through the application of a visualization score. Visualization score (VIS-A, VIS-B, or VIS-C) describes the reader’s confidence in adequate liver visualization and ability to detect liver observations, and is now recommended to be reported for all surveillance US examinations (Table 1).

**Table 1:**
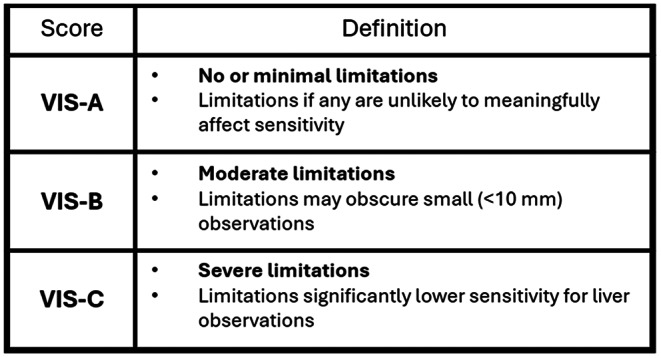
LI-RADS visulization scores

**Fig. 1:**
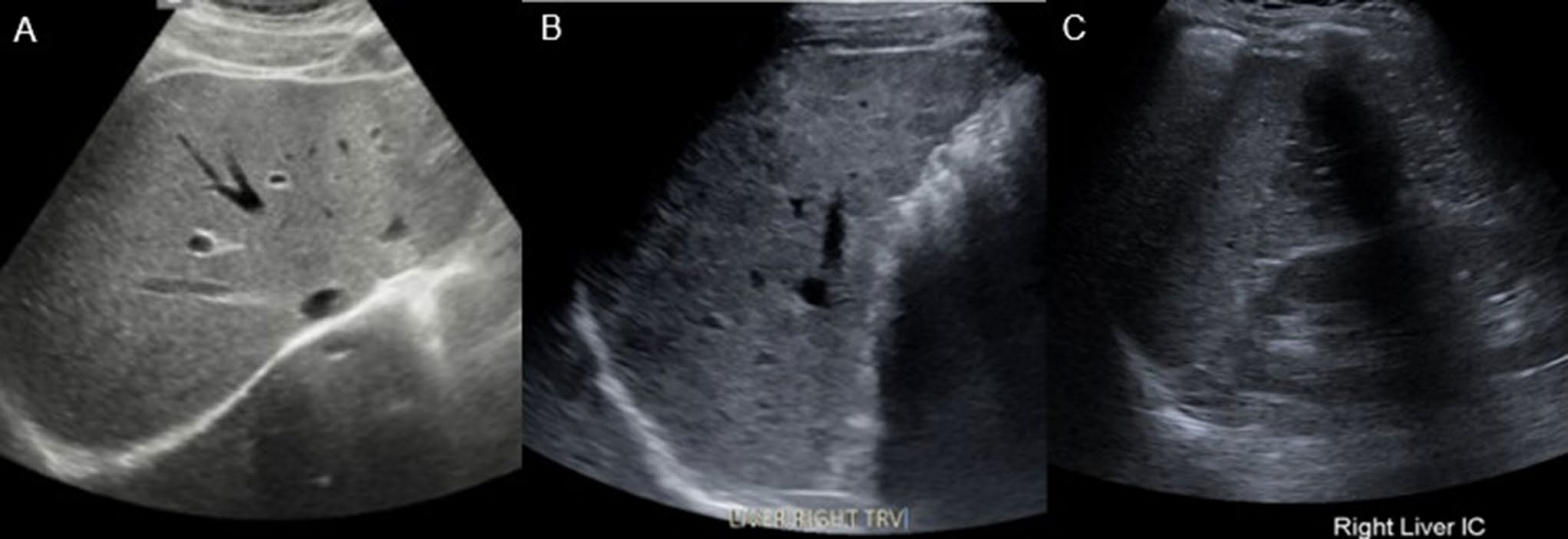
Three representative US images of the LI-RADS US Surveillance v2024 core visualization scores. **(A)** VIS-A, demonstrating no limitations in liver visualization. **(B)** VIS-B, demonstrating some limitations in parenchymal visualization due to moderate parenchymal heterogeneity. **(C)** VIS-C, demonstrating an intercostal view of a high riding liver resulting in severe limitations in parenchymal visualization due to rib shadows

A visualization score of VIS-A is applied when the entire or nearly the entire liver is well visualized and the chances of missing even sub-centimeter liver lesions is very low. VIS-B represents an exam that has moderate limitations that may obscure small (< 10 mm) observations. VIS-C should be used for exams that have significant limitations in visualization of the liver, including when large portions of one or both lobes are obscured (Fig. 1).

**Fig. 2:**
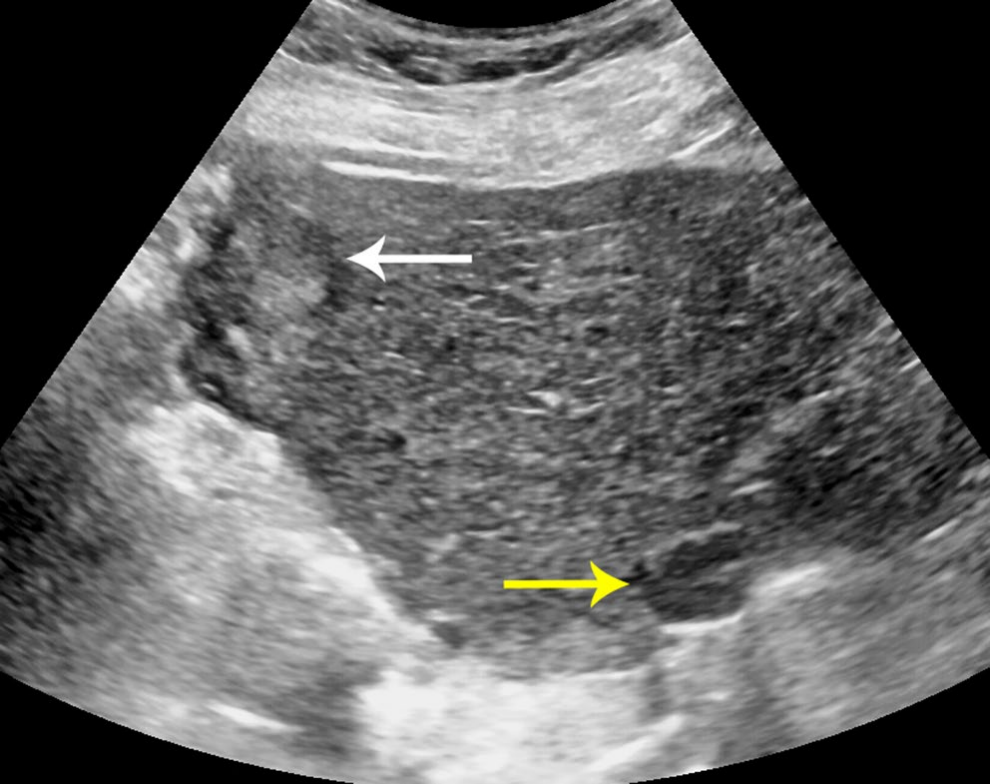
Positive surveillance US exam in a patient with HBV cirrhosis. US image demonstrates a coarsened, heterogeneous liver with otherwise no significant limitations in liver visualization, VIS-B. Two nodules (arrows), one hypoechoic and one hyperechoic, are seen. Both lesions were later confirmed to be HCC on contrast enhanced US

Currently, guidelines recommend continued use of US for surveillance of patients whose exams are scored VIS-A or VIS-B, as HCC detection sensitivity remains high (> 75%) (Fig. 2) [15].

**Fig. 3:**
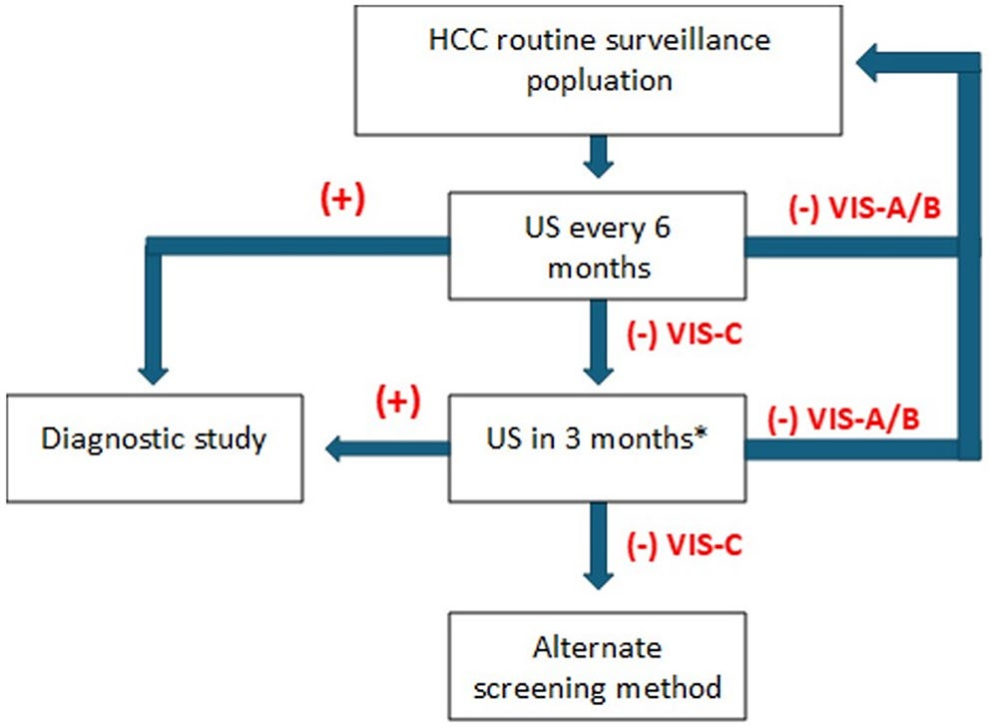
Flow chart detailing the recommended US surveillance screening algorithm utilized by LI-RADS US surveillance v2024 core. Positive results refer to observations distinct from background liver greater than 1 cm in size that are not definitely benign, including areas of parenchymal distortion or new thrombus in portal or hepatic veins. *Moving directly to an alternative screening method can be considered after first VIS-C if certain risk factors exist, including severe cirrhosis, fatty infiltration, and large body habitus

For patients with VIS-C, the recommendation is to repeat US in 3 months, as several studies have shown that > 50% of subsequent exams may improve to VIS-B or even VIS-A [11, 16]. If the repeat US exam at 3 months remains VIS-C, however, then the likelihood that the patient stays a VIS-C is greater than 50% and alternative imaging strategies are recommended. Similarly, if certain risk factors for poor visualization exist (as stated above), patients may move directly to an alternative surveillance imaging modality (Fig. 3).

This is important as VIS-C is associated with a much lower sensitivity for HCC relative to VIS-A and VIS-B scored exams (~ 27% for VIS-C vs. > 75% for VIS-A and VIS-B) [15]. One exception to this workflow is when there is an elevated or progressively increasing AFP, and no US correlate or other explanation exists. For these patients, a diagnostic multiphase CT or MRI is recommended to evaluate for a potentially US-occult HCC as the reason for the elevated AFP [6].

## Alternative imaging modalities

### Multiphasic CT and MRI

**Fig. 4:**
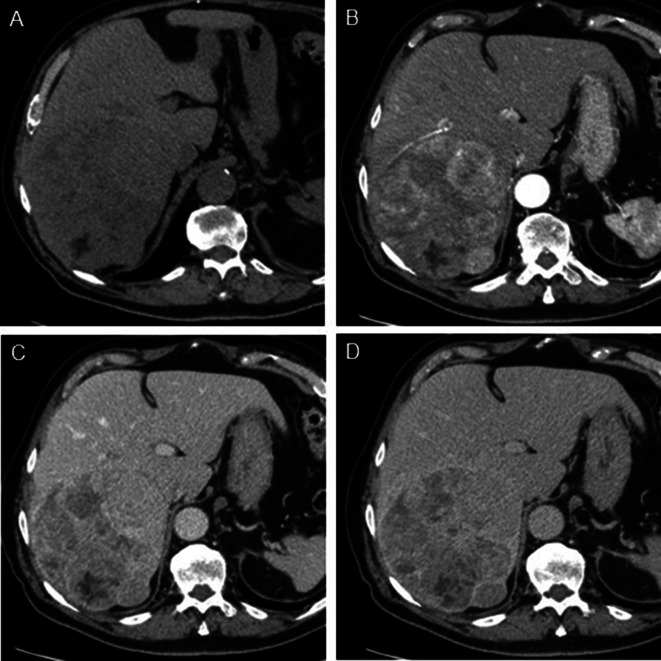
HCC on multiphasic contrast enhanced CT. Axial non-contrast **(A)**, arterial phase **(B)**, portal venous phase **(C)**, and delayed phase **(D)** post contrast CT images show a large heterogeneous right hepatic lobe mass with arterial phase hyperenhancement, washout, and enhancing capsule diagnostic of HCC

There are several options for patients in whom ultrasound is not a suitable surveillance modality, one being multiphasic contrast enhanced computed tomography (CT). Multiphase CT has been shown to have a higher sensitivity (73.6% vs. 59.3%) and positive predictive value (PPV; 85.8% vs. 77.4%) than US alone in a recent meta-analysis, again noting that the sensitivity is near equivalent when US is used in combination with serum AFP for early-stage HCC, as is recommended in the current national guidelines [14, 17] (Fig. 4).

CT also has the advantages of being fast and widely available, however, the high cost compared to US, potential risk of adverse events related to iodinated contrast, and most importantly the high cumulative radiation dose likely limits the use of CT as a general surveillance modality. CT remains a viable option when MRI is unavailable or impractical for specific patients.

**Fig. 5:**
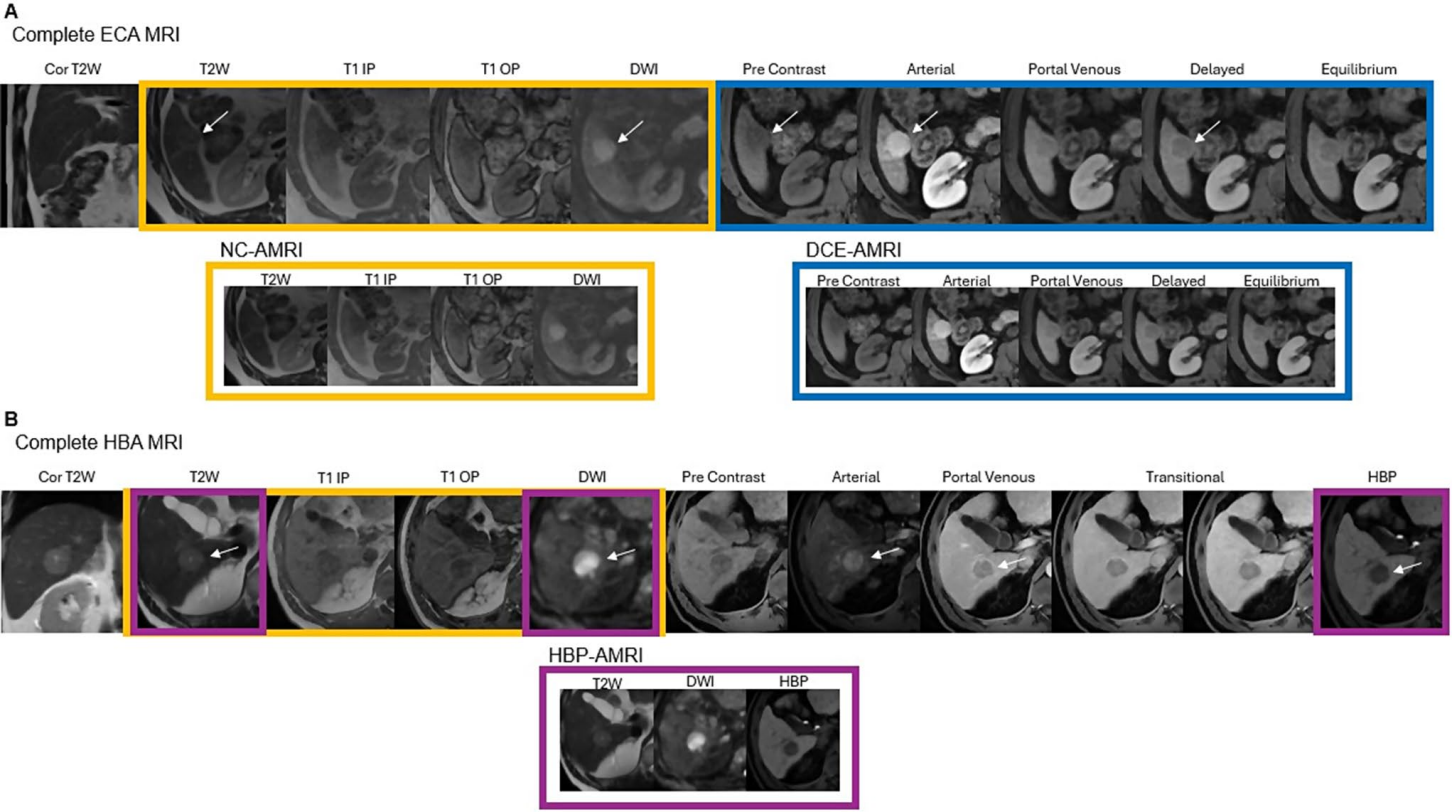
Sample multiphasic MRI and AMRI protocols in two patients with HCC. **(A)** Complete multiphasic MRI protocol utilizing an extracellular contrast agent and the select sequences utilized in the NC-AMRI and DCE-AMRI protocols. Images show a well circumscribed, partially exophytic mass (arrows) in hepatic segment 5 which is mildly T2 hyperintense, demonstrates restricted diffusion, and has arterial phase hyperenhancement, washout, and an enhancing capsule, diagnostic of HCC. **(B)** Complete multiphasic hepatobiliary agent (gadoxetic acid) enhanced MRI protocol and select sequences utilized in the HBP-AMRI protocol. Images show a well-circumscribed mass (arrows) in hepatic segment 6 which is mildly T2 hyperintense and demonstrates mild signal drop on the out-of-phase sequence as well as restricted diffusion. The mass also shows arterial phase hyperenhancement, washout, and corresponds to a hepatobiliary phase defect

MRI is often used alongside CT for the diagnosis and follow-up of HCC but has also been used for surveillance in some institutions. A multiphase liver MRI has the highest sensitivity and specificity (88% and 94% respectively) of all imaging modalities for the diagnosis of HCC, given its superior contrast resolution and the variety of sequences obtained, allowing for more nuanced problem solving and interpretation [18]. Additionally, a prospective study that evaluated gadoxetic acid-enhanced MRI vs. US in a surveillance population of cirrhotic patients found MRI to have a significantly higher detection rate of 86.0% vs. 27.9% for US with significantly fewer false positive findings than US (3.0% vs. 5.4%) [19]. Despite this, a complete multiphase MRI protocol (Fig. 5) is not a cost-effective or time-efficient surveillance method at the population level [20].

In addition to limited availability, the cost of the exam and the time required to both acquire and interpret these exams is far greater than US. Patient tolerability and safety in those with claustrophobia, inability to lie flat and/or still for long periods of time, requirement for intravenous gadolinium contrast and associated risks, and MRI incompatible implants or metallic foreign bodies are other major limitations of MRI.

### Abbreviated MRI protocols

Given the cost and access concerns for MRI-based population-level surveillance, three abbreviated MRI (AMRI) protocols have been developed including non-contrast AMRI (NC-AMRI), dynamic contrast enhanced AMRI (DCE-AMRI), and gadoxetic acid-enhanced MRI with hepatobiliary-phase imaging (HBP-AMRI). These protocols involve the acquisition of fewer sequences to reduce examination time (approximately 10–15 min, including the set-up time) and cost, without significantly compromising HCC detection rates. As with US surveillance, if an AMRI is positive, a follow-up full diagnostic multiphase examination may be required for complete work-up.

### NC-AMRI

**Fig. 6:**
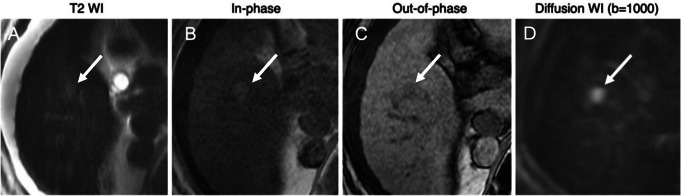
Positive surveillance NC-AMRI. Axial T2-weighted **(A)** MR image shows a subtle T2 hyperintense mass (arrow) in hepatic segment 5. The mass demonstrates iron sparing on the axial in-phase and out-of-phase T1-weigthed **(B**,** C)** MR images. Axial diffusion-weighted **(D)** MR image shows restricted diffusion within the mass, distinct from background liver parenchyma. All findings are suspicious for HCC and require confirmatory diagnostic testing

NC-AMRI includes a T2-weighted sequence, T1 in-phase and out-of-phase sequences, and diffusion weighted imaging (DWI) (Figs. 5 and 6).

This protocol avoids the potential adverse effects of intravenous gadolinium contrast and allows for easy repetition if some of the sequences are degraded by motion or another artifact. However, the lack of contrast limits the detection of some HCCs, especially early HCC, which can be nearly isointense to liver on T2-weighted and DWI sequences. Other limitations of NC-AMRI include inherent vulnerability of DWI to artifacts, need for a confirmatory test for a positive result, and limited sensitivity for detection of tumor in vein. Despite these limitations, retrospective studies have shown a per-patient sensitivity of 84–92%, noting that these studies were primarily performed in HCC enriched populations, not representative of a screening population [21–23]. One study evaluating NC-AMRI in a surveillance setting revealed a lower sensitivity of 62%, although with a specificity ranging from 96 to 100% [24]. A recent prospective multicenter study in a surveillance population revealed marginally higher sensitivity of NC-AMRI compared to US screening, but a significantly higher diagnostic yield while maintaining a comparable false referral rate [25]. In another prospective study evaluating DWI alone, sensitivity was 83% and specificity 98% for detection of HCC [26]. While this study was relatively underpowered, it does demonstrate the potential for NC-AMRI to provide a viable screening modality for patients in whom contrast is contraindicated.

### DCE-AMRI

**Fig. 7:**
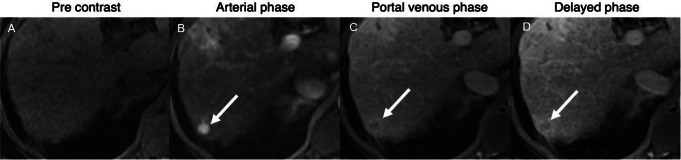
HCC on DCE-AMRI. Axial pre-contrast **(A)** and contrast-enhanced arterial phase **(B)**, portal venous phase **(C)**, and delayed phase **(D)** T1-weighted MR images show a small isointense lesion in the right hepatic lobe which demonstrates arterial phase hyperenhancement, washout, and capsule diagnostic of HCC

For patients who can receive gadolinium-based contrast agents, the DCE-AMRI and HBP-AMRI protocols are alternative surveillance options in patients with repeated VIS-C US exams. The DCE-AMRI protocol includes only pre- and dynamic post-contrast T1 weighted images using an extracellular contrast agent (Fig. 5). The advantage of this protocol is that all the major features defined in LI-RADS for diagnosis of HCC are obtainable (Fig. 7).

Therefore, a LI-RADS 5 observation identified on a DCE-AMRI still constitutes a definitive diagnosis, thus eliminating the need for an additional, complete diagnostic study. Disadvantages of this protocol include requirement for intravenous contrast and dependency on the technical adequacy of timed power injected arterial phase images. Problems with the timing and quality of the contrast bolus, patient motion, or other artifacts can render the study nondiagnostic, requiring a repeat exam. Additionally, many ancillary features cannot be assessed to upgrade or downgrade observations without supplementary sequences. Thus vascular pseudolesions, a phenomenon often seen in cirrhosis, may be over-categorized as LI-RADS 3, and may necessitate unnecessary follow-up examinations. Despite these limitations, current data suggests that DCE-AMRI may be an effective screening modality. Although studies evaluating the role of DCE-AMRI in surveillance populations or a prospective setting are still lacking, two retrospective studies completed on diagnostic cohorts with a high prevalence of HCC demonstrated comparable sensitivity and specificity of DCE-AMRI to a complete diagnostic MRI (92.1% sensitivity and 88.6% specificity) [27, 28].

### HBP-AMRI

**Fig. 8:**
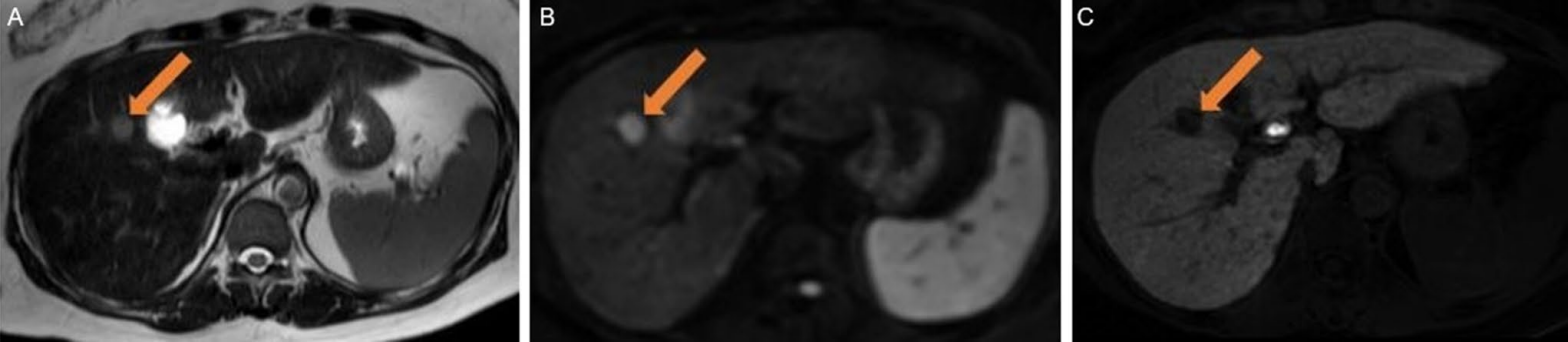
Positive surveillance HBP-AMRI. Axial T2-weighted **(A)** MR image shows a small mildly T2 hyperintense mass adjacent to the gallbladder fossa (arrow). **(B**,** C)** Axial diffusion-weighted and contrast-enhanced hepatobiliary phase MR images show that the mass has restricted diffusion and is hypointense to background liver parenchyma on hepatobiliary phase indicating the presence of non-functioning hepatocytes. HCC was confirmed on full diagnostic MRI with an extracellular contrast agent

An alternative to DCE AMRI is HBP-AMRI. This protocol includes a post contrast hepatobiliary phase acquisition obtained 15–20 min after the intravenous administration of gadoxetate disodium, a T2-weighted sequence, and DWI (Fig. 5). Gadoxetate disodium is a gadolinium-based contrast agent that has 50% clearance through the hepatobiliary system and 50% clearance through the kidneys. The OATP transporter is responsible for transporting gadoxetate into hepatocytes. HCC contains non-functioning OATP, and non-HCC malignant lesions lack the transporter [29]. Thus, while normal liver is enhanced in the HBP, HCC and other non-HCC malignant lesions are hypointense to the background liver and are thus easily detectable (Fig. 8).

In this protocol, contrast can be hand injected while the patient is in the waiting room, negating the need for a power injector, decreasing time in the MRI exam room, and simplifying technologists’ workflow. Additionally, because hepatocytes retain gadoxetate for an extended period of time, sequences can be easily repeated as necessary. At least five studies [30–34] have assessed the performance of HBP-AMRI in a surveillance setting for at-risk patients with no history of HCC. These reported a per-patient sensitivity of 80.8 − 92.0%, and specificity of 91.0 − 95.6%. A recent meta-analysis showed the pooled sensitivity and specificity of HBP-AMRI to be 86% and 94% respectively [35].

Several studies have also demonstrated a favorable cost effectiveness of HBP-AMRI. One study showed a 30% immediate cost savings relative to complete diagnostic contrast enhanced MRI [36]. Another showed a favorable incremental cost-effectiveness ratio (ICER) of $3000 per quality-adjusted life year (QALY) gained for HCC screening with HBP-AMRI versus US [37]. A recent study based on a U.S. population with MAFLD cirrhosis demonstrated that HBP-AMRI is a more cost-effective surveillance strategy in patients with visualization scores of VIS-C than US alone or no surveillance [38].

Although promising, HBP-AMRI has its limitations, most notably its limited use in patients with bilirubin level > 3 mg/dL (bilirubin outcompetes gadoxetate at the OATP transporter) and advanced cirrhosis with diminished hepatocyte uptake of contrast. Another limitation is the potential false positive results from non-HCC lesions containing non-functioning hepatocytes. Third, limited uptake of gadoxetate in areas of confluent fibrosis can obscure underlying HCC (false-negative) or be mistaken for a lesion (false-positive). Additionally, a few studies have shown that HCC’s can be iso- or hyperintense on the HBP, and may be mistaken for a benign lesion [30]. Other disadvantages of HBP-AMRI include mandatory confirmatory testing for a positive screening result, inability to characterize tumor thrombus, and the higher cost of gadoxetate.

Given the current body of evidence, all three AMRI protocols appear to be effective screening methods for patients in whom US surveillance is inadequate. As there is currently no convincing evidence to prove one protocol is superior, the decision of which protocol to use should be tailored to the individual patient and resources available. In those with normal bilirubin levels and no contraindications to gadolinium-based contrast agents, HBP-AMRI may be an ideal first choice, assuming a readily available supply of gadoxetate. DCE-AMRI can be utilized in those with elevated bilirubin levels or if gadoxetate is unavailable, but would not be a good option for patients who cannot remain still or have difficulty holding their breath. The NC-AMRI approach also remains a valid option for patients in whom gadolinium-based contrast agents are contraindicated.

## Conclusion

To conclude, US remains the most practical, well-studied, and cost-effective imaging modality for screening the vast majority of patients at risk for developing HCC. It has a proven mortality benefit and is recommended in combination with serum AFP as the first line surveillance method by the AASLD and LI-RADS. The US visualization score, as recommended in the LI-RADS US Surveillance v2024 core, supports a patient-centered surveillance strategy, helping identify those in whom US is not an effective screening method. In these subsets of patients, other surveillance modalities can be considered, particularly one of the various AMRI protocols now available.

## Data Availability

No datasets were generated or analysed during the current study.
